# 3D Printing: The Key to Success in an Otherwise Impossible Percutaneous Coronary Intervention

**DOI:** 10.1002/ccr3.71035

**Published:** 2025-10-31

**Authors:** Tim Noterdaeme, Olivier Gach, Lucien Finianos, Pieter‐Jan Palmers, Giuseppe Colletti, Claudiu Ungureanu

**Affiliations:** ^1^ Cardiovascular Departement MontLégia Liège Belgium; ^2^ Cardiovascular Departement Clinique Saint‐Joseph Arlon Belgium; ^3^ Cardiovascular Department Jolimont Hospital La Louvière Belgium

**Keywords:** 3D coronary impression, bench testing, complex coronary interventions, percutaneous coronary intervention

## Abstract

Patient‐specific 3D‐printed models could enhance PCI planning, improving feasibility and efficacy in cases where conventional methods fail or coronary anatomy is atypical. Despite their potential, adoption is limited by system availability, equipment costs, and time expenditure. Appropriate infrastructure and dedicated programs could expand and broaden their clinical application.

A 67‐year‐old female with NYHA class III dyspnea and severe coronary artery disease was referred for percutaneous coronary intervention (PCI) targeting the proximal circumflex artery. The initial procedure was aborted after multiple unsuccessful attempts to selectively engage the left main ostium during a 3‐h same session carried out successively by three different highly experienced operators (Figure 1/Video 1). Initially, we chose the guiding catheters (Extra Back‐Up EBU 3.5 and 4) based on standard clinical practice to provide an adequate balance between stability and support. As the procedure advanced and new challenges arose, we adopted a more iterative, trial‐and‐error strategy. In total, we explored ten different guiding catheter shapes using radial and femoral vascular access points. The difficulties faced in this case stemmed from two simultaneous anatomical anomalies of the left main coronary artery (LMCA). Firstly, the LMCA arose from an atypical posterior position within the aortic sinus, and secondly, it had a low takeoff, beginning nearer to the aortic valve.

**FIGURE 1:**
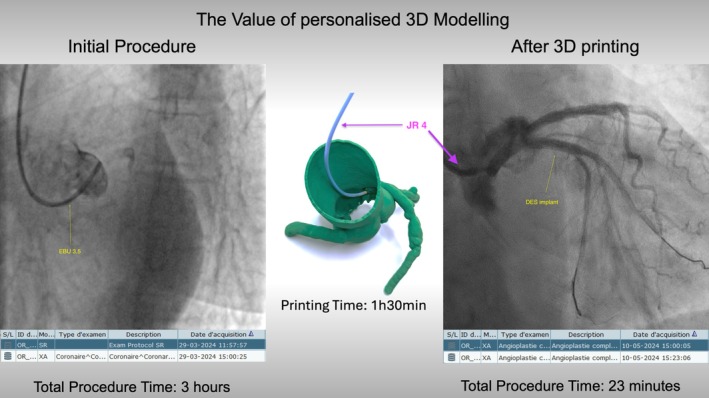
On the left side: Coronary angiogram: non‐selective contrast injection through the guiding catheter revealed difficulty in engaging the left main stem of an EBU 3,5 guide catheter. Despite attempts with 10 different shapes and types of guiding catheters, selective engagement of the left main stem could not be achieved. In the center: A 3D‐printed model of the coronary anatomy. A 1:1 scale replica helped identify the Judkins Right (JR 4) catheter as the best fit for the unique anatomy. On the right side: Coronary angiogram: optimal angiographic result after drug‐eluting stent implantation (4 × 15 mm). The bottom section: Highlights time specifications corresponding to each intervention step, allowing assessment of procedural efficiency.

**VIDEO 1:** Unsuccessful left main catheterization during index procedure. Video content can be viewed at https://onlinelibrary.wiley.com/doi/10.1002/ccr3.71035.

To better understand the patient's anatomy and select an appropriate guiding catheter, we created a detailed 3D‐printed model of the ascending aortic root and left sinus. Using coronary CT scan data, a 1:1 scale model was produced with a Bambulabs Carbon X‐1 FDM‐3D printer [1]. A specialized protocol processed the data, converting segmented files into STL format. These files were refined using 3D Slicer software and smoothed to remove any irregularities, ensuring a high level of anatomical accuracy [2]. The final model was printed using a Formlabs Form 3 printer with flexible resin, followed by UV curing and support removal, resulting in a precise 1:1 scale replica of the coronary anatomy.

Testing by a dedicated operator unexpectedly revealed that the optimal catheter for this unique anatomy was a Judkins Right catheter. A second operator then used this specific guiding catheter during a subsequent procedure, achieving successful engagement of the left main at the first attempt, as predicted by the bench test on the 3D model (Figure 1). This approach provided valuable insights, including the optimal angiographic projection for the intervention. The procedural metrics improved significantly compared to the previous attempt (Figure 1).

While the JR catheter offers less back‐up support than options like the EBU, in this specific situation, its primary curve, located near the distal tip, surprisingly allowed for easy placement of the catheter tip into the coronary ostium with minimal manipulation. This underscores the necessity of choosing a catheter that meets each case's unique anatomical features.

This article underscores the potential of utilizing a specialized protocol to create high‐quality 3D models for coronary interventions, especially in complex cases. Personalized 3D models, which can be quickly printed, offer valuable pre‐procedural insights that can enhance procedural efficiency, minimize contrast use, reduce procedure time, and ultimately lower costs. These improved metrics indicate possible clinical advantages for our patients, such as decreased procedural risks, reduced radiation exposure, and lower risk of contrast‐induced nephropathy.

## Author Contributions

**Tim Noterdaeme:** conceptualization, investigation, project administration, writing – original draft, writing – review and editing. **Olivier Gach:** supervision, validation, visualization, writing – review and editing. **Lucien Finianos:** validation, writing – review and editing. **Pieter‐Jan Palmers:** supervision, writing – review and editing. **Giuseppe Colletti:** writing – review and editing. **Claudiu Ungureanu:** conceptualization, supervision, writing – review and editing.

## Consent

Written informed consent was obtained from the patient for the publication of this case report and any accompanying images.

## Conflicts of Interest

The authors declare no conflicts of interest.

## Data Availability

Research data are not shared.
